# Impact of Shopping Tourism for the Retail Trade as a Strategy for the Local Development of Cities

**DOI:** 10.3389/fpsyg.2020.00067

**Published:** 2020-01-31

**Authors:** Ana Isabel Muro-Rodríguez, Israel Roberto Pérez-Jiménez, Jesús Antonio Sánchez-Araque

**Affiliations:** ^1^Econometrics Area, Department of Spanish and International Economy, Econometrics, History and Economic Institutions. University of Castilla-La Mancha, Ciudad Real, Spain; ^2^Department of Business Administration. University of Castilla-La Mancha, Ciudad Real, Spain

**Keywords:** tourism and retail, shopping tourism, tourist behavior, differentiation of tourist destinations, local development

## Introduction

Shopping Tourism is a recent concept that is defined as a contemporary form of tourism carried out by individuals for whom the acquisition of goods, outside their place of residence, is a determining factor in their decision to travel (WTO, 2014, p.13).

Shopping tourism is currently one of the main strategic lines of countries to promote sustainable and quality tourism (Yu and Littrell, 2003; Tosun et al., 2007; WTO, 2014, 2017, 2018; The Shopping Quality Tourism Institute, 2015, 2017; Tourspain, 2015, 2018). This tourism is the one that generates wealth and distributes it equitably, is environmentally sustainable and has a positive impact on the environment (The Shopping Quality Tourism Institute, 2015, 2017; MINCOTUR, 2019b). It implies an important economic and employment impact for the cities and countries in which it takes place, and favors deseasonalization and diversification (The Shopping Quality Tourism Institute, 2015, 2017).

The impact that this commercial tourism has on employment and economic growth in the countries is considerable, making it a key strategic industry (Jansen-Verbeke, 1991; Yu and Littrell, 2003; Tugcu, 2014; Webster and Ivanov, 2014; Tourspain, 2015; Albayrak et al., 2016).

Spain has become one of the most important tourist powers in the world (Tourspain, 2018). In 2017, it was the second country in the world in number of tourists and in income from international tourism with nearly 87,000 million euros (WTO, 2018; MINCOTUR, 2019b). Then in 2018 the tourism sector closed with a new record of 82.6 million international tourists and a volume of 89,678 million euros (MINCOTUR, 2019a).

Given these figures, it is necessary to take advantage of this potential market and bet on the quality and diversification of the offer of tourism products, as a key element of the National and Integral Tourism Plan, and this will be achieved by promoting quality tourism and shopping as an engine of economic and social growth (Tourspain, 2015).

This strategic line of exploiting the relationship between trade and tourism and taking advantage of the existing synergies between the two (Getz, 1993; Ryan et al., 1999; Global Blue, 2015), can help the city retail sector to adapt to new consumption habits. Also, it can open new horizons and avoid the disappearance of city centers as places of commerce due to the closure of businesses (Tourspain, 2015; MINECO, 2017). Additionally, the tourism sector can avoid its main weaknesses such as the typical tourist offer of the sun and beach, the concentration of demand in time and territory, or the strong dependence of certain issuing countries (Tourspain, 2015; MINCOTUR, 2019b).

This tourism is an increasingly attractive alternative for improving local commercial activity and, at the same time, shopping can be an active potential for the city to differentiate itself as a chosen destination (Tourspain, 2015; The Shopping Quality Tourism Institute, 2017; MINCOTUR, 2019b).

In this sense, there are different specific initiatives to promote cities as shopping tourism destinations, both in the city center and for other areas, most of which are centered on Madrid and Barcelona, since both occupy the second position among the best European cities as shopping destinations (Travé, 2019). In order to establish strategies, destinations must have a high level of knowledge about the socio-economic profile of tourists, their main motivations for traveling and the choice of destination (Žabkar et al., 2010; Choi et al., 2016a), especially because this knowledge can influence the competitive capacity of tourist destinations as shopping destinations (Kim et al., 2011; Cohen et al., 2014).

The aim of this research is to highlight the importance of shopping tourism for cities: (1) need for the commercial and tourism sectors to exploit the interrelationships between the two, (2) socio-economic impact on local trade, (3) identify differentiation strategies for cities as shopping destinations and (4) establish recommendations based on the analysis of key factors for listing a city as a shopping destination.

## Shopping Tourism and its Impact on Cities

Shopping as a tourist activity has recently been analyzed. Purchasing tourism has received little attention so there are few publications in this area (Choi et al., 2016b).

Shopping is a common and fundamental tourist activity both from the tourist's perspective and for the chosen destination (Jin et al., 2017). It is one of the most important activities for tourists (Yu and Littrell, 2003; Lloyd et al., 2011; Albayrak et al., 2016). In many cases, shopping is an important factor in the choice of destination (Moscardo, 2004) and in others, it is may be the main reason for traveling (Timothy, 2005; Lehto et al., 2014).

Therefore, it is very important to know on what attributes tourists base their decisions (Dann, 1981; Žabkar et al., 2010; Hult et al., 2017) and what valuation purchases have on their trips (Murphy et al., 2000; Oh et al., 2004; Wong and Wan, 2013). Since 2000, researchers have shown an increased interest in examining people's motives for shopping while traveling and how they are influenced by the tourist destination (Albayrak et al., 2016; Choi et al., 2016a).

From a tourist perspective, Albayrak et al. (2016) and Jin et al. (2017) show most studies that deal with the relationships between sociodemographic characteristics and purchasing attitudes (Kim and Littrell, 2001; Yazdani, 2007; Yüksel and Yüksel, 2007; Alegre and Cladera, 2012), purchasing motivation (Law and Au, 2000; Swanson and Horridge, 2006; Yüksel, 2007; Kattiyapornpong and Miller, 2012); consumer satisfaction (Heung and Cheng, 2000; Reisinger and Turner, 2002; Wong and Law, 2003; Chang et al., 2006; Tosun et al., 2007; Murphy et al., 2011a,b; Wong and Wan, 2013; Chang, 2014), shopping characteristics (Choi et al., 2008; Han et al., 2015), outdoor environment and procurement evaluation (Christiansen and Snepenger, 2002; Yüksel, 2004, 2013).

For cities, shopping can be an important source of income (Murphy et al., 2011a,b). Shopping tourism also favors the creation of job opportunities and improves the image of cities as tourist destinations (Cohen et al., 2014; Tugcu, 2014; Webster and Ivanov, 2014). In this sense, many cities use tourism shopping as a promotional strategy to differentiate themselves from the competition (Ryan et al., 1999; Snepenger et al., 2003; Coles, 2004; Rabbiosi, 2011, 2015; Hurst and Niehm, 2012; Timothy, 2014).

It must be kept in mind that in the consumer's choice not only the physical attributes of a product count. For the maximization of the usefulness of the tourist's behavior there are other attributes like visiting places and spaces where to understand the local culture and strengthen social bonds (Timothy, 2005; Saayman and Saayman, 2012). These variables are relevant for differentiating cities in terms of their attributes.

Better information on the patterns and factors that condition the behavior of shopping tourists offers opportunities to the cities' tourism industry (Jansen-Verbeke, 1991). On the one hand, it allows for better planning and management of sales and expenses (Timothy, 2014) and on the other hand, it serves as a basis for promoting alternative places of attraction, creation of spaces and brand development of a city (Kemperman et al., 2009; Rabbiosi, 2011, 2015). Thus a new strategic line emerges for areas that can base their attractiveness on shopping tourism (Murphy et al., 2011b). They are generally cities or areas with a pleasant environment marked by historical or natural features along tourist routes, in tourist destination areas or near urban centers. However, they differ from urban businesses and shopping districts because they have a specialized retail trade and a distinctive atmosphere (Getz, 1993). These relationships between shopping and tourism are based on the study of literature and some empirical results that relate shopping as a means of tourist attraction (Jansen-Verbeke, 1991, 2000).

It is important to analyze advantages and disadvantages when talking about sustainable development and quality tourism. Social and environmental costs must be balanced with economic benefits (Ryan et al., 1999; Moscardo and Murphy, 2014; The Shopping Quality Tourism Institute, 2017). Tourism is a very important sector for the achievement of Sustainable Development Goals 2030 proposed by the United Nations. The World Tourism Organization links tourism to the 17 objectives of sustainable development (WTO, 2016).

The sustainable development of tourism consists of making optimal use of the environmental resources that are a fundamental element of tourism development, maintaining essential ecological processes and helping to conserve natural resources and biological diversity (WTO, 2014). This concept is developed through five strategic axes: collaborative governance, sustainable growth, tourism space, companies and people, product, marketing and tourism intelligence, and competitive transformation (MINCOTUR, 2019b).

The competitive transformation of tourism is linked to the concept of Intelligent Tourism Systems (SIT) for the promotion of Intelligent Tourist Destinations (SEGITTUR, 2015) and the concept of Tourism 4.0. Tourism 4.0 is a term that has been mentioned since 2017 to refer to tourism that encourages technological changes in tourism. These changes in the tourism industry range from the digitization of establishments using the Internet to offer a service to everything related to interactions through mobile devices such as maps, GPS, information about shops, restaurants, etc. (Zupan, 2019). Examples of these strategies have been developed in Palma de Mallorca, Las Palmas de Gran Canaria, Badajoz and the island of El Hierro, considered the first smart island in the world. Las Palmas de Gran Canaria stands out with the implementation of the first model to promote shopping tourism in the city through multilingual mobile technology (SEGITTUR, 2015). In this context, different strategic questions arise regarding the socio-economic impact of shopping tourism that influence local and sustainable development (Jin et al., 2017).

Outstanding as local development strategies are “Town Centre Management” focused on downtown management models (Coca-Stefaniak et al., 2009) and the Business Improvement Districts (BIDs) used to revitalize the urban centers of British cities such as Birmingham, Plymouth and for the promotion of Tourist Shopping Villages (TSV) (Getz, 1993; Murphy et al., 2011a,b). According to the World Tourism Organization, cities as shopping destinations must take into account accessibility, available infrastructure, security, cleanliness, attractive location, destination marketing and promotion, connection with the tourism value chain, research and development, regulation and adequate training and education (WTO, 2014).

All these strategies condition the choice of tourist destination, so a collaboration is necessary between all the businessmen agents (commercial companies, transport, accommodation), tourism promoters and local institutions. In this sense, the joint participation of all agents to position Spain, or certain cities, as a destination for shopping tourism is very important.

## Discussion

Tourism is an increasingly attractive alternative for improving local commercial activity and, at the same time, retail trade can be an active potential for the city to differentiate itself as a chosen destination (Tourspain, 2015). With proper planning, tourism can be a part of the solution to cities' economic problems by helping to avoid closing businesses in cities that accelerate city depopulation problems and cause economic losses for families' economies. For this reason, the concept of tourist purchases (WTO, 2014; Tourspain, 2015), gains importance and is defined as an activity in which tourists buy goods during their trip (Jin et al., 2017).

In order for cities to take advantage of the synergies between tourism and trade, it is necessary to analyze the different local development strategies that can be implemented such as: “Town Centre Management” (Coca-Stefaniak et al., 2009), the Business Improvement Districts (BIDs) and the “Tourist Shopping Villages” (TSV) (Getz, 1993; Murphy et al., 2011a,b); and all the variables that cities must have in mind to differentiate themselves as destinations (WTO, 2014; SEGITTUR, 2015).

Given all the variables necessary to promote a city as a tourist shopping destination, it is necessary to establish joint strategies with the participation of all local agents, such as traders and their associations, and local institutions to establish strategies to position cities internationally as a tourist shopping destination, relating to the commercial management of retail establishments in Spain and aimed at promoting quality and sustainable shopping tourism, as one of the strategic lines to boost the competitiveness of the sector (SEGITTUR, 2015; Tourspain, 2015; MINECO, 2017; MINCOTUR, 2019b).

## Author Contributions

AM-R, IP-J, and JS-A contributed conception of the opinion article. AM-R wrote the first draft of the manuscript. IP-J and JS-A performed the state of the art and the conceptual framework. All authors contributed to manuscript revision, read and approved the submitted version.

### Conflict of Interest

The authors declare that the research was conducted in the absence of any commercial or financial relationships that could be construed as a potential conflict of interest.
